# Determination of plasma concentrations of levofloxacin by high performance liquid chromatography for use at a multidrug-resistant tuberculosis hospital in Tanzania

**DOI:** 10.1371/journal.pone.0170663

**Published:** 2017-01-31

**Authors:** Andrew Ebers, Suzanne Stroup, Stellah Mpagama, Riziki Kisonga, Isaack Lekule, Jie Liu, Scott Heysell

**Affiliations:** 1 Division of Infectious Diseases and International Health, University of Virginia, Charlottesville, United States of America; 2 Kibong’oto Infectious Disease Hospital, Kilimanjaro, Tanzania; Institute of Immunology and Experimental Therapy, Polish Academy of Sciences, POLAND

## Abstract

Therapeutic drug monitoring may improve multidrug-resistant tuberculosis (MDR-TB) treatment outcomes. Levofloxacin demonstrates significant individual pharmacokinetic variability. Thus, we sought to develop and validate a high-performance liquid chromatography (HPLC) method with ultraviolet (UV) detection for levofloxacin in patients on MDR-TB treatment. The HPLC-UV method is based on a solid phase extraction (SPE) and a direct injection into the HPLC system. The limit of quantification was 0.25 μg/mL, and the assay was linear over the concentration range of 0.25—15 μg/mL (y = 0.5668x—0.0603, R^2^ = 0.9992) for the determination of levofloxacin in plasma. The HPLC-UV methodology achieved excellent accuracy and reproducibility along a clinically meaningful range. The intra-assay RSD% of low, medium, and high quality control samples (QC) were 1.93, 2.44, and 1.90, respectively, while the inter-assay RSD% were 3.74, 5.65, and 3.30, respectively. The mean recovery was 96.84%. This method was then utilized to measure levofloxacin concentrations from patients’ plasma samples from a retrospective cohort of consecutive enrolled subjects treated for MDR-TB at the national TB hospital in Tanzania during 5/3/2013–8/31/2015. Plasma was collected at 2 hours after levofloxacin administration, the time of estimated peak concentration (eC_max_) treatment. Forty-one MDR-TB patients had plasma available and 39 had traceable programmatic outcomes. Only 13 (32%) patients had any plasma concentration that reached the lower range of the expected literature derived C_max_ with the median eC_max_ being 5.86 (3.33–9.08 μg/ml). Using Classification and Regression Tree analysis, an eC_max_ ≥7.55 μg/mL was identified as the threshold which best predicted cure. Analyzing this CART derived threshold on treatment outcome, the time to sputum culture conversion was 38.3 ± 22.7 days vs. 47.8 ± 26.5 days (p = 0.27) and a greater proportion were cured, in 10 out of 15 (66.7%) vs. 6 out of 18 (33.3%) (p = 0.06) respectively. Furthermore, one patient with an eC_max_/minimum inhibitory concentration (MIC) of only 1.13 acquired extensively drug resistant (XDR)-TB while undergoing treatment. The individual variability of levofloxacin concentrations in MDR-TB patients from Tanzania supports further study of the application of onsite therapeutic drug monitoring and MIC testing.

## Introduction

Multidrug-resistant tuberculosis (MDR-TB), defined as resistance to both isoniazid and rifampin, has inferior treatment outcomes when compared to drug-susceptible TB in part because second-line medications have lower potency, higher side effect profiles and require longer durations of treatment [1–2]. While low plasma concentrations of anti-TB drugs have been associated with poor treatment outcomes in drug-susceptible TB [3], such data for agents used in MDR-TB treatment are more limited. We have previously shown among patients being treated with a standardized MDR-TB regimen in Tanzania that plasma drug activity as measured by an *in vitro* assay was driven by the concentrations relative to the minimum inhibitory concentrations (MICs) of kanamycin and levofloxacin [4–5]. As there was significant individual pharmacokinetic variability, we subsequently demonstrated that increases in plasma drug activity during the early weeks of treatment predicted a favorable treatment outcome [5–6]. Unfortunately, the *in vitro* assay does not quantify specific drug concentrations that would be necessary for dose adjustment. Yet these findings suggested that routine measurement of drug concentrations, termed therapeutic drug monitoring (TDM), could be of programmatic benefit.

In other settings, TDM has been be used to hasten culture conversion, prevent acquired drug resistance and limit therapy induced toxicity, but is not routinely performed in TB-endemic locations because of the need for technical expertise and the cost of chromatography or mass spectrometry [7–8]. Nevertheless, MDR-TB is often managed in partnership with specialized centers or reference laboratories, as is the practice at Kibong’oto Infectious Diseases Hospital, the national TB referral hospital and the affiliated Kilimanjaro Clinical Research Institute in Northern Tanzania. We therefore endeavored to design a high-performance liquid chromatography (HPLC) assay to be used onsite for the detection of levofloxacin plasma concentrations.

Levofloxacin has potent activity against *Mycobacterium tuberculosis* both in vitro and in vivo [7], is the fluoroquinolone within the standardized MDR-TB regimen in Tanzania, and was determined to be more suitable to dose adjustment than kanamycin given the greater pharmacokinetic variability with oral administration and previous studies demonstrating MICs closer to the critical concentration of resistance. Area under the concentration curve (AUC)/MIC measurements remain the preferred predictor of drug activity, but it is practically difficult to obtain frequent blood samples from ill patients in our setting [8]. However, a plasma draw at 2 hour (C_2hr_), does correlate to the peak concentration (C_max_) for fluoroquinolones and thus may serve as a useful estimate of concentration dependent drug activity. Suggested target ranges for C_max_ of 8 to 12 μg/ml have been proposed [8]. However, one recent study demonstrated that levofloxacin doses in the range of 17 to 20 mg/kg were associated with good target attainment for MICs from 0.25 to 0.50 μg/ml while an MIC of 2.0 μg/ml were associated to poor target attainment across all doses, but this study, like many, used population ranges of MIC [7]. Our prior studies have suggested that most patients in Tanzania were being dosed with levofloxacin at less than 17 mg/kg and it was hypothesized that many would have MICs between 0.5 and 2.0 μg/ml [4–5].

Thus, the following study describes the development and assay performance of a HPLC method with ultraviolent (UV) detection for levofloxacin in human plasma. We then tested the assay in plasma from patients undergoing MDR-TB treatment in Tanzania with well-described treatment outcomes to characterize further the estimated peak concentrations (eC_max_) of levofloxacin relative to the patient’s own *M*. *tuberculosis* MIC and the suggested target ranges in the literature.

## Materials and methods

### Solid phase extraction (SPE) protocol

Levofloxacin (LFX) and phenacetin (PHN, internal standard (IS)) were purchased from Sigma–Aldrich (St. Louis, MO, USA). The purity of all the standard drugs was ≥99%. HPLC grade methanol, acetonitrile, water and formic acid were purchased from Fisher Scientific (Pittsburgh, USA). 500 μL of frozen patient plasma was allowed to thaw before being combined with 500 μL of 4% phosphoric acid. A prepared stock solution of phenacetin (internal standard) was prepared and added to the plasma sample. Levofloxacin and phenacetin were extracted from plasma by SPE using Oasis HLB Extraction Cartridges (30 μm) (manufacturer Waters, Milford, MA). Cartridges were conditioned with methanol and water prior to the addition of 500 μL of plasma sample followed by two washes with 10% methanol and 5% NH_4_OH respectively, then subsequently eluted with 2% formic acid in 90% methanol. The eluents were evaporated and the residue reconstituted in 100 μL of 5% acetonitrile (S1 Fig).

### HPLC conditions

Separation by HPLC was achieved by injecting 8 μL onto a C18 column (Acclaim 120) at 30°C with mobile phase of continuous acetonitrile gradient of 5–75% with 10mM-monobasic potassium phosphate of pH 3.5 (1.0 mL/min) and processed within the Dionex UltiMate 3000 HPLC (Thermo Scientific, Waltham, USA). Optimum detection for levofloxacin and PHN was at 295 nm and 260 nm respectively. Data was acquired and chromatograms were analyzed with Chromeleon 7.2 software (S1 Fig).

### Study subjects, data collection

Medical charts from patients who underwent MDR-TB treatment were reviewed for demographic and clinical data. Patients were considered eligible for inclusion if they had a *M*. *tuberculosis* isolate with resistance to isoniazid and rifampin and had been started on a MDR-TB regimen containing levofloxacin. Patients were excluded if they had extrapulmonary TB. Patient pretreatment characteristics and historical information were collected, including: age, gender, pretreatment weight, height, pretreatment chest x-ray cavities, percentage of lung involved, history of cigarette smoking, history of alcohol use, history of previous TB, and number of prior TB episodes. Per hospital protocol, all patients are tested for HIV and if positive, a CD4 count (cells/mm^3^) is measured for all patients not already on antiretroviral therapy (ART). ART is started within 8 weeks of MDR-TB treatment initiation. The first-line ART regimens were tenofovir, emtricitabine and efavirenz; or zidovudine, lamivudine and efavirenz.

A retrospective cohort of consecutive enrolled subjects treated for MDR-TB at the national TB hospital in Tanzania during 5/3/2013–8/31/2015 had plasma sampled at 2 and 4 weeks after starting levofloxacin. A 750 mg oral dose daily was given to all patients as part of the standardized MDR-TB regimen. Other drugs within the regimen included kanamycin given as a 15-mg/kg intramuscular dose, cycloserine given as a 500-mg oral dose, ethionamide given as a 250-mg oral dose, and pyrazinamide (PZA) given as a 20- to 30-mg/kg daily oral dose. On the morning of plasma sampling, medications were given while the patient was fasting and blood drawn at 2 hours after directly observed administration for C_2hr_, or hereafter, the eC_max_. Plasma was then immediately frozen and stored at -80°C until batch analysis on the Dionex UltiMate 3000 HPLC.

### Drug susceptibility and treatment monitoring

Per hospital protocol, one of the patient’s pretreatment isolates was sent to the national reference laboratory in Dar es Salaam for confirmation of first-line drug susceptibility testing and second-line susceptibility testing to ofloxacin and kanamycin by the agar proportion method on a case-by-case basis. If available, the patient’s pretreatment isolate was also tested for ofloxacin MIC on MYCOTB Sensititre plate (TREK Diagnostics, Cleveland, USA) at the Kilimanjaro Clinical Research Institute along with 11 other TB drugs. Ofloxacin MIC was used as a proxy for levofloxacin as it was not present on the plate. The MICs reported were not available for therapeutic decisions.

Sputum was collected for smear microscopy and culture on Lowenstein-Jensen (LJ) solid agar prior to treatment initiation and monthly thereafter until completion of the intensive phase. The intensive phase was at least 8 months in duration and at least 6 months beyond the date of sputum culture conversion. Culture conversion was defined as the date of the first of two negative cultures separated by at least one month without later relapse of culture positivity. Following the intensive phase, kanamycin was discontinued and the patient was discharged to complete at least 20 total months of oral therapy with the monitoring of decentralized treatment facilities throughout the country.

Upon completion of treatment, outcomes were determined. Death was categorized as due to any cause; default, if treatment was interrupted for greater than eight weeks; failed treatment, if acquired drug resistance developed or lack of sustained sputum culture conversion to negative; cure, disease-free and without relapse; and treatment completed, if disease-free and relapse could not be confirmed. Written consent was obtained for all patients. If a patient was not literate, consent was explained verbally to both the patient and a literate surrogate who was able to sign the consent paperwork. The institutional review boards at Kilimanjaro Christian Medical College, National Institute for Medical Research (Tanzania) and the University of Virginia approved the above consent procedure and the study.

### Statistical analysis

Statistical analysis was performed using SPSS 23.0 (IBM, Chicago, Illinois, USA). Possible correlations between pharmacokinetic parameters were assessed including: age, sex, mg/kg levofloxacin dose, and HIV status. For group comparison the Student’s t-tests was applied for normally distributed variables, and the Kruskal–Wallis and Mann Whitney U test for non-normally distributed variables. A two-tailed p value of 0.05 was considered significant. Classification and Regression Tree (CART) analysis was used to analyze the impact of levofloxacin levels on the treatment outcome using R statistical software (http://r-project.org) and Data Mining using Rattle (http://rattle.togaware.com) [3].

## Results

### HPLC protocol and validation

**Fig 1:**
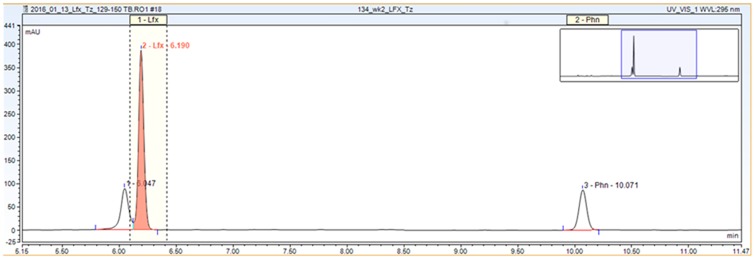
Representative HPLC-UV chromatogram for levofloxacin with phenacetin (Internal standard). Representative Chromatogram for Levofloxacin.

A five-point calibration was constructed for levofloxacin. Calibration standards spanning the 0.25–15 μg/mL range were made up based on the known weight of levofloxacin spiked into 1 ml plasma. Internal standard calibration was used with the analytical signal based on the corrected peak area obtained from the integration. The assay was linear over the concentration range of 0.25—15 μg/mL with a LOD of 0.25 μg/ml (y = 0.5668x—0.0603, R^2^ = 0.9992) for the determination of levofloxacin in plasma (S1 Fig). The upper limit of quantification in plasma was 20 μg/mL. Intra-day and inter-day precision were 1.90–2.44%RSD and 3.30–5.65%RSD, respectively (Table 1) showing excellent repeatability and reproducibility. Further comparison with referral lab samples was excellent across spiked concentrations of 0.625–40 μg/mL, National Jewish Health, Denver, CO (S1 Fig). A peak adjacent to the primary levofloxacin peak was noted on some chromatograms and likely represents the enantiomer of levofloxacin that developed in the presence of sample preparation conditions that favored the transition to its racemic form, dextrofloxacin, (Fig 1). Thus, the method was felt to be adequate for direct analysis of levofloxacin in plasma from patient samples.

**Table 1:** Inter-assay variability of calibration curve, Intra-day precision and Inter-day precision.

Nominal concentration (μg/ml) n = 3 series/day, 2 days				
Levofloxacin:	LQC^a^	MQC	HQC	Phenacetin
**μg/mL:**	1	4	10	mAU
**Intra-day precision (n = 10)**				
**Mean, SD**	1.08 ± 0.02	3.97 ± 0.10	10.78 ± 0.20	89.70 ± 1.82
**RSD %**	1.93	2.44	1.90	2.03
**Inter-day precision**^b^ **(n = 20)**				
**Mean, SD**	1.10 ± 0.04	4.11 ± 0.23	10.88 ± 0.36	85.32 ± 5.77
**RSD %**	3.74	5.65	3.30	6.76

^a^ Low quality control (LQC), Medium quality control (MQC), High quality control (HQC). RSD = relative standard deviation.

^b^ Samples analyzed over 5 days

### Baseline patient characteristics

Forty-one patients had plasma available for levofloxacin concentration measurements, and pretreatment characteristics are shown in Table 2. The mean age was 37.9 (± 12.2 years). Mean body weight was 49.0 (± 10.5 kg) and the mean body mass index was 18.6 (± 3.1 kg·m^-2^). Within this cohort, 16 (38.1%) were co-infected with HIV and the mean CD4 count was 273.4 (± 165.3) cells/ml. Of those patients with HIV, 8 (50%) were on ART prior to initiation of MDR-TB treatment. Thirty-five (85.3%) patients had complete clinical information regarding the number of prior episodes of TB, and 24 of these (68.5%) had two or more episodes of TB prior to their diagnosis of MDR-TB. Chest radiography demonstrated a mean number of 1.5 (± 2.1) cavitary lesions.

**Table 2:** Patient characteristics.

Characteristics	n = 41
Age, years	37.9 ± 12.2
Sex, males	29 (71.4)
Substance use	
Alcohol	19 (45.7)
Tobacco	12 (29.7)
HIV Positive	16 (38.1)
CD4 count in cells/ml	273.4 ± 165.3
Taking Antiretroviral therapy	8 (50)
Prior TB Episodes ^a^	
Zero	4 (11.4)
One	7 (20.0)
Two	24 (68.5)
Pretreatment Weight in kg	49.0 ± 10.5
Levofloxacin mg/kg dose	16.0 ± 3.6
Body Mass Index in kg·m^-2^	18.6 ± 3.1
Levofloxacin mg/BMI dose	41.5 ± 7.0
Percentage of Lung Involved	37.6 ± 21.5
Number of Cavities	
Mean (SD)	1.5 ± 2.1
Median MIC (IQR)	
Ofloxacin^b^	1.0 (0.5–1.0)
Kanamycin	1.2 (1.2–1.2)
Cycloserine	8.0 (8.0–16.0)
Ethionamide	1.2 (1.2–20.0)

Data are presented as mean and standard deviation or as numbers (%) unless otherwise specified.

^a^ 35 patients had historical data regarding prior TB episodes

^b^ Ofloxacin MIC testing was performed in 30 patients

Pretreatment *M*. *tuberculosis* isolates for 30 (73.2%) patients were available for MIC testing to second-line drugs. It was observed that the median MIC to ofloxacin was 1.0 (0.5–1.0 μg/ml) with a minimum of 0.25 μg/ml and a maximum of 4.0 μg/ml. Additionally, for 13 isolates, sequencing for *gyr*A mutation was available from a separate study [9], and despite MICs near the breakpoint of resistance, all were found to be wildtype. In contrast, MICs to kanamycin were tightly clustered around a median MIC of 1.20 μg/ml without any demonstrating MICs near the breakpoint for resistance.

### Levofloxacin eC_max_ distribution

**Fig 2:**
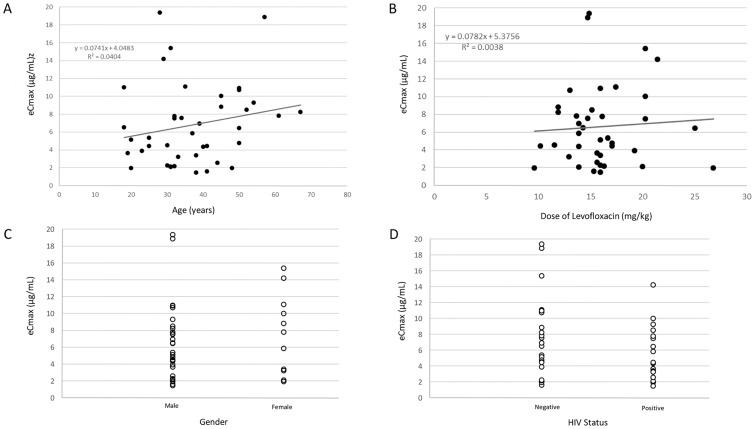
Distributions of plasma concentrations of levofloxacin by pretreatment characteristics. A) eCmax lack of correlation with Age [R2 0.0404] B) eCmax lack of correlation with mg/kg levofloxacin dose [R2 0.0038] C) eCmax by Gender; mean eCmax for Males was 6.64 and Females 7.18, p = 0.74 D) eCmax by HIV Status; mean eCmax for those who were Positive was 5.94 and Negative 7.35, p = 0.34

Overall, the median levofloxacin eC_max_ for all patients was found to be 5.86 (3.33–9.08 μg/ml). There was no significant difference between eC_max_ at 2 weeks, median 4.42 (2.26–7.83 μg/ml), compared to 4 weeks, median 5.14 (2.58–8.85 μg/ml), p = 0.62. Importantly, there was no adequate predictor of eC_max_ by gender, age, HIV status, and mg/kg dose (Fig 2). Among those patients with isolates available for MIC testing for ofloxacin (n = 30), using the highest recorded reading of eC_max_ (between the 2 week and 4 week measurements), the median eC_max_/MIC was 6.51 (2.67–19.1).

### Literature expected levofloxacin C_max_ and outcomes

Only 13 of 41 (31.3%) patients had an eC_max_ greater than the literature suggested lower limit, C_max_ of 8 μg/ml, for levofloxacin. Of the 41 patients, 2 were lost to follow-up and 39 had available programmatic treatment outcomes. Favorable outcomes included cure in 16 (41%) and treatment completion 10 (25.6%). Unfavorable outcomes included default in 6 (15.4%), death in 6 (15.4%), and acquired drug resistance in 1 (2.6%).

Those with eC_max_ below and above the literature suggested lower limit C_max_ had a mean time to sputum culture conversion in days of 45.6 ± 25.3 vs 39.2 ± 25.4, p = 0.49, respectively. Less were cured when the eCmax was below the literature suggested lower limit, 9/26 (34.6%) vs 7/13 (53.8%), although this difference did not reach statistical significance, p = 0.25. CART analyses was completed with cure as the dependent variable. CART analyses was conducted to identify a peak concentration as a predictor of cure as this parameter is commonly used for clinical care [3]. The identified predictor for cure was a levofloxacin level of 7.55 μg/mL. Analysis of treatment outcome by this CART derived threshold are shown in Table 3.

**Table 3:** CART derived eC_max_ and outcomes.

	eC_max_ < 7.55 μg/ml	eC_max_ ≥ 7.55 μg/ml	P value
	n = 18	n = 15	
Time to Sputum Culture Conversion in days	47.8 ± 26.5	38.3 ± 22.7	0.27
Treatment outcome^a^			
Cured	6 (33.3)	10 (66.7)	0.06
Treatment completed	7 (38.9)	3 (20.0)	
Death	4 (22.2)	2 (13.3)	
Development of acquired drug resistance	1 (4.5)	0 (0)	
Favorable^b^	13 (72.2)	13 (86.7)	0.31
Unfavorable^c^	5 (27.8)	2 (13.3)	

Data are presented as mean and standard deviations or as numbers (%)

^a^ 2 patients were lost to the system and do not have treatment outcomes. Default patients were excluded from analysis

^b^ A favorable outcome was defined as Cured or Treatment Completed.

^c^ An unfavorable outcome was defined as death and development of acquired drug resistance

Analysis of the 10 patients with the lowest eC_max_/MIC ratio revealed a mean eC_max_/MIC of 2.18 (± 0.92), and included one patient with eC_max_/MIC of 0.83 at 2 weeks and 1.13 at 4 weeks that acquired further resistance to levofloxacin and kanamycin, extensively drug-resistant (XDR)-TB, while on treatment. The patient’s pre-treatment isolate was susceptible to ofloxacin and kanamycin at the national referral lab, and MIC changes from the pre and during treatment isolates have been previously reported and were notable the changing in ofloxacin MIC from 4.0 to 8.0 μg/ml and kanamycin MIC from 1.2 to 20.0 μg/ml [10].

## Discussion

While TDM of first-line agents has been investigated in a number of studies [11–17], evaluation of TDM for second-line TB drugs has been limited to a small number of agents or has utilized mass spectrometry which may be cost-restrictive for most TB endemic settings [18–23]. Quantitative methods for levofloxacin have been described with HPLC-UV [24], but these studies have not applied such techniques to patient plasma for validation. Here, we describe the successful development and validation of HPLC-UV for rapid and accurate determination of levofloxacin concentrations in plasma from patients across the clinically relevant range. Given the assay’s precision, accuracy, and lower limit of detection, we believe it is applicable for TDM of levofloxacin for our patient population undergoing MDR-TB treatment in Tanzania.

TDM may be particularly important given our findings that no pretreatment clinical characteristic was able to predict *in vivo* drug exposure. Pharmacokinetic variability was common, with only 13 patients (31.3%) achieving a plasma eC_max_ above the lower limit of the literature-derived expected range. These findings are similar to our previous study of patients on the standardized MDR-TB regimen in Tanzania [5]. Importantly, the majority of patients for whom their *M*. *tuberculosis* isolate was available for testing had an ofloxacin MIC between 0.5 μg/ml and 2.0 μg/ml, where 2.0 μg/ml is the concentration used for determining resistance. These patients remain at significant risk of failing to attain AUC/MIC targets that have been predictive of efficacy in other studies, even when concentration peaks are above the lower limit of the expected range (8 μg/ml) [25]. This may be less consequential for non MDR-TB where first-line drugs have wild-type MICs that are usually much lower than the concentration used for resistance determination, but even with rifampin, pharmacokinetic variability alone has driven clinical and microbiological outcomes [3,26]. Hence for the fluoroquinolones, such differences in MIC could be even more impactful, and would not be detected by standard phenotypic testing that uses only a single concentration for a dichotomous read-out of susceptibility or resistance. Furthermore, empiric dose increase of levofloxacin without TDM will improve drug exposure, but that strategy may risk unnecessary dose-related toxicities in a patient with a *M*. *tuberculosis* isolate that has a very low MIC.

We did not necessarily expect that analysis of a single drug eC_max_ without the concentrations of other drugs in the regimen would predict outcomes, yet some observations are worth noting. While the majority of patients (66%) had a favorable outcome of cure or treatment completion comparable to other cohorts from TB endemic settings [27], patients with eC_max_ of levofloxacin below the expected range had a longer time to culture conversion, and when these results were dichotomized using a CART derived threshold of an eC_max_ <7.55 μg/ml, those subjects had a lower proportion of cure. Furthermore, the patient with the lowest levofloxacin eC_max_/MIC acquired drug resistance to both levofloxacin and kanamycin. Nevertheless, some subjects with “low” eC_max_ of levofloxacin had a favorable outcome which may be due to adequate drug exposure of the companion drugs in the regimen, delayed absorption whereby adequate AUC was preserved, or other host factors. Certainly, using a single eC_max_ value to define adequate levofloxacin exposure oversimplifies a complex dynamic [28], particularly when such values may be derived from other diverse TB-infected populations or healthy controls. Therefore, it appears increasingly relevant to define population-specific AUC/MIC thresholds using more biologically apt non-linear methods such as CART for all key drugs at which a “therapeutic floor” exists.

The study has several limitations given the retrospective design of the plasma sampling which was primarily employed for the HPLC-UV assay development and validation. For example, it is known that conventional drug-susceptibility testing does correlate with MDR-TB treatment outcome [29], yet a considerable proportion of the study population was prescribed pyrazinamide without confirmation of susceptibility, per local standard of care. Patients with pyrazinamide-resistant *M*. *tuberculosis* may have been more likely to have poor outcome despite adequate levofloxacin exposure in this population. We have since undertaken a larger prospective study to define AUC/MIC thresholds for all drugs in the MDR-TB regimen to quantify the impact of each on treatment outcome. AUC/MIC thresholds could lead to dose adjustment of individual drugs within the regimen, a clinically actionable parameter, that may optimize cure and reduce toxicity. As mentioned, the lack of blood draws in the dosing interval while not feasible for defining an actual AUC, could have better assessed delayed absorption which may have accounted for why some patients with lower levofloxacin eC_max_ were cured. Prospective study of limited sampling strategies, which may represent a more feasible method of obtaining a more complete set of PK data in TB endemic settings, and use of dried capillary blood spots on filter paper or other non-blood samples, which simplify PK sample storage until analysis, could be of further benefit [7, 30].

## Conclusion

The HPLC-UV assay performed well across a range of clinically relevant concentrations. In patients treated for MDR-TB in Tanzania, pharmacokinetic variability for levofloxacin was common and MICs were near the borderline of resistance. Variability was not predicted by mg/kg dosing or other common clinical factors, and the majority had estimated peak concentrations below the expected range. TDM appears to be an actionable tool that when coupled with MIC testing can provide the patient with an individualized dose and regimen to give the best chance of cure, even within a standardized formulary.

## Supporting Information

S1 Data Set Patient Demographics and Pharmacokinetic data. (SAV)

S1 Fig Solid phase extraction protocol, HPLC conditions and validation (accuracy, precision, linearity, inter-day precision, intraday precision. (DOCX)
