# Supplementary material for: Determination of plasma concentrations of levofloxacin by high performance liquid chromatography for use at a multidrug-resistant tuberculosis hospital in Tanzania
Source: PLoS One. 2017 Jan 31;12(1):e0170663. doi: 10.1371/journal.pone.0170663 (PMC5283651; doi:10.1371/journal.pone.0170663)

LFX Serum or Plasma Drug Level Detection by HPLC

**A. Purpose**

Therapeutic drug monitoring in multidrug-resistant tuberculosis (MDR-TB) endemic settings may aid in the treatment of patients in that the pharmacokinetic variability for fluoroquinolones is thought to contribute to poor treatment outcomes.

**B. Materials**

The following are needed for storage, serum or plasma sample preparation by SPE, analysis by HPLC.

Vortex

Freezer -80°C

Boxes dark storage (USA Scientific 2350-5809)

Pipettes (5-20 µl, 20-200 µl and 100-1000 µl)

Pipet tips (pipet tips with aerosol barriers for preventing cross-contamination are recommended)

Micro-centrifuge tubes 2.0 ml O-ring PP graduated screw cap (Sarstedt 72.694.406)

Tubes 15 ml (Corning 430053)

Adapters (Sigma 57020-U)

Syringe 5 mL (Fisher 033772)

Oasis HLB 3cc (60mg) Extraction Cartridges (SPE) (column) (Waters WAT094226)

HPLC Water (Fisher W6-4)

Methanol (MeOH) (Fisher A452-1)

Phosphoric Acid (Fisher A242-4)

Acetonitrile (ACN) (Fisher BP2405SK-4)

Ammonium hydroxide (NH4OH) solution 10% in Water (Fluka 17837-1L; Fisher)

Formic acid ≥ 99.5% LCMS 2mL Ampule (Fisher A1172AMP) (store at 4 ºC in the dark)

Phenacetin (Sigma PHR1094-1G)

HPLC Vial Clear 350 µL 100PK (Fisher C4000-LV1W)

HPLC Vial Cap with preslit T/S septa (Fisher 5000-75C)

Thermo Scientific™ Dionex™ UltiMate 3000™ system

7272976 SR‐3000 (without degasser)

8108694 Pump LPG‐3400SD

8108533 Autosampler WPS‐3000TSL (analytical)

6007113 Column Compartment TCC‐3000SD

8108468 Photometer DAD‐3000

Thermo Scientific™ Acclaim 120 C18 HPLC column, Dionex Bonded Silica Reversed Phase (Fisher 059148/20522741)

**C. IMPORTANT NOTES**

- **For all steps, allow the fluid to move through the column by gravity; once all fluid has passed through the column matrix, proceed with the next step. If necessary, use syringe and adapter to aid flow through.**
- **All water is HPLC water.**
- **Formic acid is aliquoted into HPLC vials (330 µL, least amount of air space), stored in dark storage boxes at 4 ºC.**
- **Phenacetin stock solution is aliquoted into HPLC vials (330 µL, least amount of air space), stored in dark storage boxes at 4 ºC.**
- **Add the Phenacetin internal control to the serum or plasma samples prior to the addition of the phosphoric acid.**

**D. Preparation of Phenacetin (internal control)**

Weigh out 0.01g (10 mg) into a glass dram vial and add 1 mL of 100% acetonitrile for a “stock solution” of 10 mg/mL.

Prepare a “working solution” (1 µg/µL), diluting the stock solution 1:10 by adding 10 µL of stock solution to 90 µL of HPLC water.

Add 2 µL (2 µg) of the Phenacetin “working solution” to each of the 500 µL serum or plasma samples to be extracted by SPE.

**E. Preparation of Standard Controls**

Calibration curve

A calibration curve is prepared weekly in the same biological matrix as the samples, serum or plasma, in the intended study by spiking the matrix with known concentrations of the analyte.

The number of standards used in constructing the calibration curve was a function of the anticipated range of analytical values and the nature of the analyte / response relationship.

Calibration curves were constructed using spiked serum or plasma samples over the concentration ranges of 0.25–15 µg/mL. Linear calibration graphs were plotted as the ratio of peak area of the analyte to peak area of IS vs the respective analyte concentration at ten levels of 0.25, 0.5, 1.0, 2.0, 4.0, 6.0, 8.0, 10.0, 12.0, 15.0 mcg/mL for LFX.

Concentrations of standards were chosen on the basis of the concentration range expected in the particular study.

A calibration curve consisted of a blank sample (matrix sample processed without internal standard), a zero sample (matrix sample processed with internal standard), and six to eight non-zero samples covering the expected range, including Lower Limit of Quantification (LLOQ).

**F. Procedure**

Remove serum or plasma samples (500 µL aliquots) from -80 ºC freezers and allow thawing in the dark (place in drawer).

Place labeled column into a 15 ml corning tube.

Add 2 µL (2 µg) of Phenacetin “working solution” to each of the 500 µL serum or plasma samples to be extracted by SPE.

Condition the column by adding 1 mL of 100% Methanol (MeOH).

Add 500 μL 0f 4% phosphoric acid to each of the 500 μL serum or plasma samples; pulse vortex to mix;

Incubate in the dark (drawer) at room temperature for a 5-25 mins.

Equilibrate the column by adding 1 mL HPLC water.

Load the 1 mL of treated sample (500 μL serum or plasma + 500 μL 4% phosphoric acid) onto the column.

Wash 1: add 1 mL of wash1: 10% MeOH in water.

Wash 2: add 1 mL of wash 2: 5% Ammonium hydroxide (NH4OH) in 10:90 MeOH: H2O.

**Preparation of wash 2 solution:**

| Total volume (mL) | HPLC Water (mL) | MeOH (mL) | 10% NH4OH in Water (mL) |
| --- | --- | --- | --- |
| 4 | 1.6 | 0.4 (400 µL) | 2 |
| 8 | 3.2 | 0.8 (800 µL) | 4 |
| 35 | 14 | 3.5 | 17.5 |
| 50 | 20 | 5 | 25 |

Push the remaining wash 2 solution through the column with the adapter/syringe.

Place a 2.0 ml Sarstedt tube (elute collection tube) into a clean 15 ml tube.

Place the column into the 15 ml tube that has the Sarstedt collection tube present.

Elute: add 2 x 500 μL of elution solution: 2% Formic acid (HCOOH) 90:10 MeOH: H2O.

**Add the first aliquot of 500 µL of elution solution to each column immediately after removing the remaining wash 2 solution by the adapter/syringe.**

**If necessary, aid the first aliquot of 500 µL of elution solution into the column by gentle use of the adapter/syringe.**

**Preparation of elution solution:**

| Total volume (mL) | HPLC Water (mL) | MeOH (mL) | 99.9% Formic Acid (mL) |
| --- | --- | --- | --- |
| 4 | 0.32 (320 µL) | 3.6 | 0.08 (80 µL) |
| 8 | 0.64 (640 µL) | 7.2 | 0.16 (160 µL) |
| 35 | 2.8 | 31.5 | 0.7 (700 µL) |
| 50 | 4 | 45 | 1 |

Push the remaining elution solution through the column and into the collection tube with the adapter/syringe.

Dry samples with the use of a speed vacuum.

Reconstitute the dried Oasis Extraction Cartridge eluates with 100 μL of 5% acetonitrile in water; may store samples at 4 ºC overnight for HPLC rum next day.

Without dilution, inject 8 μL of the 100 μL directly onto the HPLC C18 column for analysis.

**G. HPLC Run Conditions**

HPLC: Thermo Scientific™ Dionex™ UltiMate 3000™ system

Column: Acclaim 120 (Thermo Fisher), C18, 5 μm particle size, 120 Angstrom pore size;

Dimensions 4.6 x 150 mm, Reversed Phase

Solvent A: 10 mM potassium phosphate monobasic pH 3.5

Solvent B: 70% acetonitrile: 30% 10 mM potassium phosphate monobasic pH 3.5

Elution Gradient: Continuous acetonitrile gradient of 5% - 75% B

(Before injection, equilibrate for 7 min. B)

Temperature: 10°C samples; 30°C column

Flow rate: 1.0 ml/min

Injection volume: 8 µL

Wavelength: UV, 295 nm for 8.5 mins (levofloxacin, LFX); then 260 nm for 2.5 mins (phenacetin, PHN)

**H. Analytics**

Systematic computational analysis of data was performed by internal instrument software, specifically Thermo Scientific™ Dionex™ CHROMELEON™7.2.3.7553 Chromatography Data System (CDS) software.

Results reported in µg/mL using Chromeleon CDS and integration tools, Cobra™ peak detection wizard and SmartPeaks™ Integration Assistant, to enhance the accuracy of the data processing method.

**I. Validation protocol for accuracy, precision, linearity, inter-day precision, intraday precision**

A five-point calibration was constructed for levofloxacin. Calibration standards spanning the 0.25 - 15 µg/mL range were made up based on the known weight of levofloxacin spiked into 1 ml plasma. External standard calibration was used with the analytical signal based on the corrected peak area obtained from the integration. Each calibration standard was analyzed in triplicate. Inter-assay variability of calibration curve on five consecutive days showed a linear and reproducible curve in the observed analytical ranges.

The assay was linear over the concentration range of 0.25 - 15 µg/mL with a LOD of 0.25 µg/ml and a LLOQ of 0.25 µg/ml (y = 0.5668x – 0.0603, R2= 0.9992) for the determination of levofloxacin in plasma. The upper limit of quantification in plasma is 20 µg/mL. Intra-day and inter-day precision were 1.90 – 2.44 %RSD and 3.30 – 5.65 %RSD, respectively, showing excellent repeatability and reproducibility.

Inter- and intra-day precision and accuracy were determined in five consecutive days (n=20) and on one day (n=10), respectively, at LLQC ( 0.25 µg/ml of LFX), LQC (1.0 µg/ml of LFX), MQC (4.0 µg/ml of LFX) and HQC (10.0 µg/ml of LFX) concentration levels. Peak area responses obtained from chromatograms were used to graph calibration curves and estimate accuracy and precision. Accuracy was evaluated as the percentage ratio of nominal concentration to mean measured concentration. Precision was determined from percent relative standard deviation (%RSD) at the mean measured concentration.


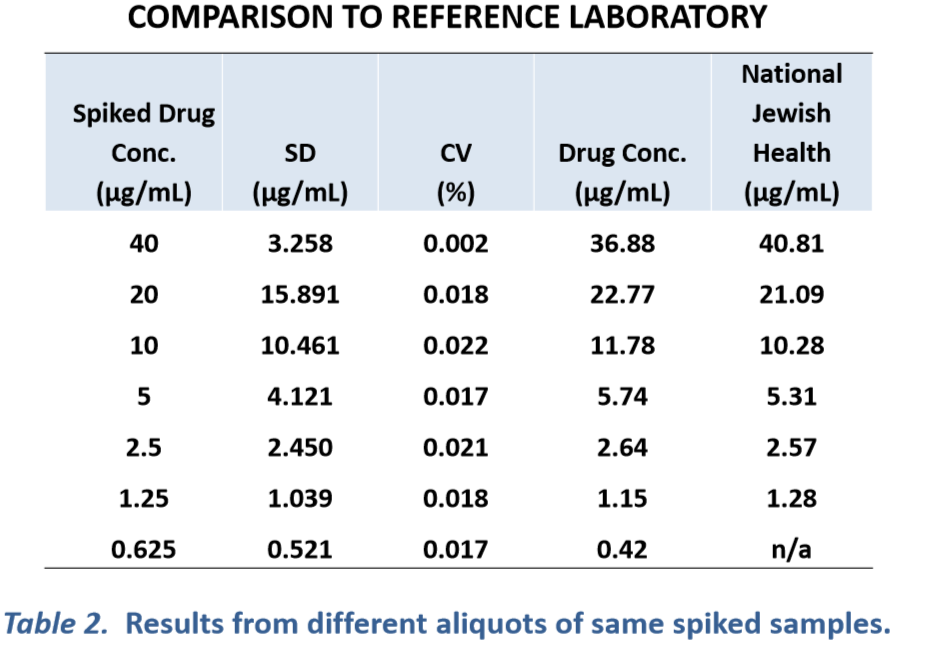

Supplement: S1 Fig — (DOCX) [file pone.0170663.s002.docx]
